# Prediction of cervical stromal invasion using ultrasound radiomics: from conventional ultrasound to intelligent diagnosis

**DOI:** 10.3389/fonc.2026.1817583

**Published:** 2026-05-07

**Authors:** Xiaoli Peng, Qisen Zhu, Lu Zhao, Ruyun Li, Ling Tu, Jiao Chen, Guocheng Du, Maochun Zhang

**Affiliations:** 1 Department of Ultrasound, Affiliated Hospital of North Sichuan Medical College, Nanchong, Sichuan, China; 2 Department of Thyroid and Breast Surgery, Nanchong Central Hospital, North Sichuan Medical College, Nanchong, Sichuan, China; 3 Department of Health Management Center, Affiliated Hospital of North Sichuan Medical College, Nanchong, Sichuan, China

**Keywords:** artificial intelligence, cervical stromal invasion, endometrial cancer, radiomics, ultrasound

## Abstract

**Rationale and objectives:**

Accurate preoperative assessment of cervical stromal invasion (CSI) in endometrial cancer (EC) is crucial for surgical planning, but conventional imaging has limited diagnostic performance. This study aims to develop and validate a non-invasive ultrasound radiomics model to improve the preoperative prediction accuracy of CSI.

**Materials and methods:**

We retrospectively analyzed 294 patients with pathologically confirmed EC, randomly assigned (7:3) to training (n=205) and test (n=89) cohorts. Regions of interest were manually segmented on 2D ultrasound images using 3D-Slicer, from which 837 radiomics features were extracted. Following screening for reproducibility (ICCs ≥ 0.75) and univariate analysis, the features were finally reduced to 25 via LASSO regression, and the radiomic score (Radscore) was calculated. Similarly, six clinical predictors (menopause duration, parity, CA-125, tumor size, endometrial-myometrial junction, vascularity grade) were identified via univariate analysis and LASSO regression and combined into a clinical score (C_score). Logistic regression was used to build radiomics, clinical, and combined nomogram models. Performance was assessed using AUC, calibration curves, and decision curve analysis (DCA).

**Results:**

The radiomics model achieved the highest AUCs: 0.975 (95% CI: 0.958–0.991) in the training cohort and 0.906 (95% CI: 0.848–0.965) in the test cohort, significantly outperforming the clinical model (AUC: 0.947 and 0.832) and the nomogram model (AUC: 0.945 and 0.838). Furthermore, it showed good calibration and provided substantial net clinical benefit across a wide range of threshold probabilities on DCA.

**Conclusion:**

Ultrasound radiomics is a promising non-invasive tool for preoperatively predicting CSI in EC, with potential to enhance personalized treatment planning.

## 1 Introduction

Endometrial cancer(EC) has now become one of the most common gynecological malignancies among women, with its incidence rates rising globally, although ovarian cancer remains the leading cause of death from gynecologic malignancy, EC poses a significant health burden due to its increasing prevalence (1, 2). GLOBOCAN 2022 estimates indicate roughly 417,000 incident cases worldwide, representing 4.5% of female malignancies, and approximately 97,000 deaths (3). In developed countries such as the United States, the incidence of EC has even surpassed that of cervical cancer, posing a serious threat to women’s health (4). The lifetime risk of developing EC is 3.1%, with an overall five-year survival rate of 81%. The median age at diagnosis is 64 years. When localized disease is diagnosed and surgically removed, the five-year survival rate can reach as high as 95%. However, for patients with distant metastatic disease, the five-year survival rate drops significantly to only 18% (3, 5).According to the international federation of gynecology and obstetrics (FIGO) staging system (2023), stage II EC is defined as tumor invasion of the cervical stroma without extension beyond the uterus (6). Multiple studies have shown that cervical stromal invasion (CSI) is associated with reduced patient survival rates, and deep (exceeding one-third) cervical stromal involvement increases the risk of lymph node metastasis (7, 8). CSI is an independent prognostic factor for mortality in FIGO stage II EC patients (9). The national comprehensive cancer network (NCCN) guidelines recommend cervical biopsy or pelvic magnetic resonance imaging (MRI) for patients with suspected or grossly visible cervical involvement. Patients with negative findings should undergo total hysterectomy with bilateral salpingo-oophorectomy and surgical staging, whereas those with positive findings should receive total hysterectomy or radical hysterectomy, bilateral salpingo-oophorectomy, and surgical staging. Radical hysterectomy is particularly indicated when negative surgical margins are required. The 5-year survival rate for FIGO stage I EC is 88%, which decreases to 75% for stage II (10, 11).This indicates that the presence and accurate assessment of cervical stromal involvement hold significant clinical implications for determining surgical approaches and adjuvant treatment strategies, as well as for predicting survival outcomes and prognosis in EC. In recent years, the development of radiomics has provided new insights into the evaluation and management of EC. By enabling high-throughput extraction and analysis of features from ultrasound images, radiomics transforms imaging data into quantifiable information (12). Lin Y et al. (13) developed a radiomics model using diffusion-weighted imaging (DWI) to predict high risk of lymph node metastasis or recurrence in patients with EC. Chen Y et al. (14) developed a multimodal deep learning(DL) radiomics model based on MRI for preoperative differentiation of myometrial invasion in EC. In addition, numerous studies have demonstrated that radiomics can be applied to the diagnosis (15, 16) and preoperative guidance (17), as well as prognosis prediction (18) in EC. Transvaginal ultrasound (TVUS) is the most commonly used preoperative screening modality for EC, providing a wealth of analyzable images. It is cost-effective, allows real-time dynamic assessment, is non-ionizing, and offers high repeatability. Liu X et al. (19)and Wang Q et al. (20)developed radiomics models based on color doppler ultrasound to predict lymph node metastasis in EC. Li YX et al. (21) established a machine learning(ML) model based on ultrasound to predict the risk of EC in postmenopausal women. These studies collectively demonstrate the feasibility of using ultrasound images for radiomic analysis in EC. While several studies have shown that MRI-based radiomics models can predict CSI in EC patients (22, 23), research on ultrasound radiomics for this specific purpose remains scarce. Therefore, this study aims to develop a radiomics model using transvaginal ultrasound images to predict CSI in EC, potentially providing valuable guidance for clinical decision-making.

## 2 Materials and methods

### 2.1 Study population

**Figure 1:**
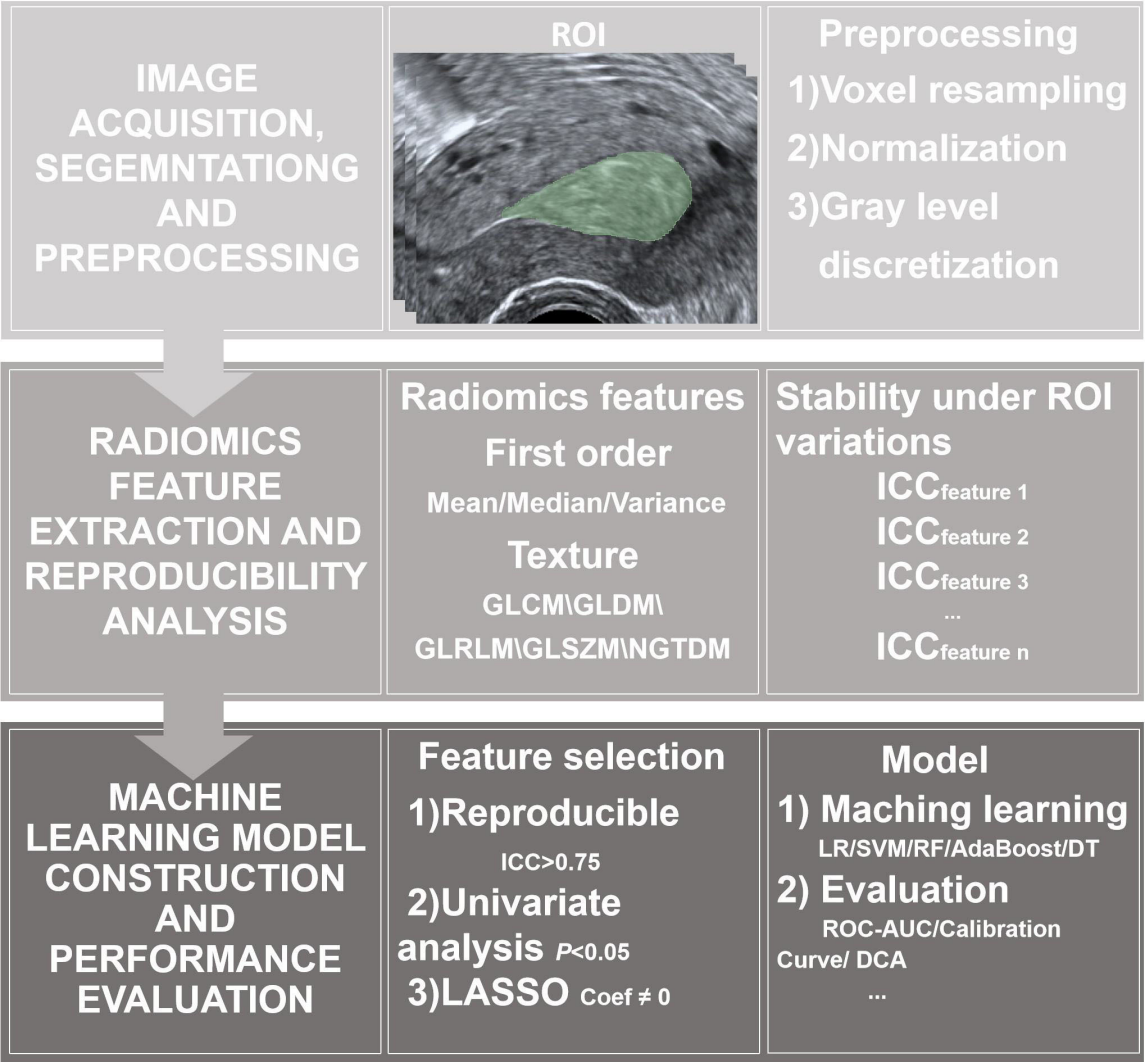
Flowchart of the study process. AdaBoost, gradient boosting; DCA, decision curve analysis; DT, decision trees; GLCM, gray-level co-occurrence matrix; GLDM, gray-level dependence matrix; GLRLM, gray-level run length matrix; GLSZM, gray-level size zone matrix; ICC, intraclass correlation coefficient; LR, logistic regression; NGTDM, neighboring gray tone difference matrix; ROI, regions of interest; RF, random forests; ROC-AUC, the area under the receiver operating characteristic curve; SVM, support vector machines.

A retrospective analysis was conducted on 294 patients with pathologically confirmed EC treated at our institution from 2018 to 2024. Inclusion criteria were: ① definitive surgical pathology diagnosis of EC; ② absence of other endometrial lesions; ③ preoperative ultrasound examination performed at our hospital. Exclusion criteria included: ① concurrent malignancies; ② prior local or systemic treatments such as radiotherapy, chemotherapy, or surgical resection before the ultrasound examination; ③ poor image quality affecting judgment; ④ incomplete pathological or medical records. A total of 294 female patients were included, aged between 32 and 82 years, with a mean age of 54.65 ± 8.641 years, all of whom were married. Among them, 119 patients had CSI, while 175 did not. The detailed study flowchart is shown in **Figure 1**. This study was approved by the ethics committee of our institution (approval number: 2025ER408-1) and in compliance with the provisions of the declaration of Helsinki. Informed consent was waived due to the retrospective nature of the study.

### 2.2 Instruments and methods

Ultrasound examinations were performed using GE Voluson E8 and E10 ultrasound systems equipped with transvaginal probes (frequency range: 3.0–9.0 MHz). Patients were instructed to empty their bladders prior to the examination. They were positioned in the dorsal lithotomy position, and the uterus, adnexa, and pelvic cavity were scanned continuously. The lesion was examined using multiple scanning planes, and images were stored in DICOM format in the picture archiving and communication system (PACS). Ultrasound findings, including uterine size and lesion diameter, were recorded for each patient. Two-dimensional (2D) grayscale images depicting the lesion in the plane of maximum diameter were selected for subsequent analysis.

### 2.3 Collection of clinical and pathological characteristics

Clinical and pathological data were retrospectively retrieved from the institutional electronic medical records system. The collected variables encompassed demographic information, medical history, laboratory results, and detailed ultrasound findings. Specifically, the following parameters were recorded: age; presence of vaginal bleeding or discharge; menstrual disturbances; abdominal pain; post-coital bleeding; comorbid conditions including pelvic inflammatory disease, atherosclerosis, hypertension, diabetes, hyperlipidemia, fatty liver, and heart disease; obstetric and gynecological history (prior cesarean section, ectopic pregnancy, previous uterine surgery, age at menarche, age at menopause, duration of menopause, menopausal status, age at marriage, gravidity, parity, and number of abortions or induced abortions); anthropometric measurements (weight, height, and body mass index [BMI]); serum tumor markers (carcinoembryonic antigen [CEA], carbohydrate antigen 125 [CA-125], squamous cell carcinoma antigen [SCC-Ag], and cytokeratin 19 fragment [CYFRA 21-1]); human papillomavirus (HPV) status; and ultrasound characteristics including uterine volume, uterine morphology, presence of intrauterine fluid collection, maximum tumor diameter, tumor echogenicity (homogeneity and pattern), tumor shape, integrity of the endometrial-myometrial junction, tumor vascularity grade (assessed via color Doppler), and endometrial thickness.

### 2.4 Tumor segmentation

Prior to segmentation, the selected 2D ultrasound images were processed using a standardized interpolation protocol. Although the analysis was performed on a single two-dimensional plane, we adopted the 3D-Slicer software’s default isotropic resampling setting (1 mm × 1 mm) to ensure that the in-plane resolution (x and y axes) was harmonized to 1 mm × 1 mm. This standardization was performed to eliminate variability caused by different original pixel spacings in the raw ultrasound data and to ensure the stability of the radiomic feature calculations. Subsequently, the gray-level intensities of all images were normalized. Two physicians blinded to the pathological results—a senior radiologist and an attending radiologist—manually delineated the regions of interest (ROIs) along the tumor margins using 3D-Slicer software (version 5.5.0). In cases of disagreement, consensus was reached through discussion, resulting in a final contour for each lesion (**Figure 1**). To assess reproducibility, 50 randomly selected cases were re-contoured by both physicians one month after the initial segmentation. The intra- and inter-observer reproducibility of the tumor segmentation was evaluated using the intraclass and interclass correlation coefficients (ICCs).

### 2.5 Feature extraction and selection

Feature extraction was performed using the radiomics package integrated within the 3D-Slicer software (version 5.5.0). Features with intra- and inter-observer intraclass correlation coefficient (ICCs) ≥ 0.75 were retained to ensure high reproducibility. The extracted features were standardized using Z-score normalization. Feature selection was conducted using t-tests or Mann-Whitney U tests, followed by least absolute shrinkage and selection operator (LASSO) regression to identify significant predictors. To ensure rigorous model validation and prevent data leakage, the 10-fold cross-validation process during LASSO regression was strictly performed within the training set.

### 2.6 Model construction

To reduce the dimensionality of the radiomic features and prevent overfitting, a two-step feature selection process was employed. Crucially, to avoid data leakage, all feature selection procedures were strictly performed exclusively within the training dataset. First, univariate analysis (e.g., t-test or Mann-Whitney U test) was conducted on the training set to filter out features that showed no significant difference between groups. Subsequently, the LASSO regression algorithm was applied to the remaining features in the training set to further select the most valuable predictive features. The final model was then constructed based on these selected features and evaluated in the test set. Various ML algorithms, including logistic regression (LR), support vector machines (SVM), random forests(RF), gradient boosting(AdaBoost), and decision trees(DT), were utilized to construct radiomic models.

For hyperparameter tuning:

SVM: A grid search was performed to optimize the gamma (range: 10–^3^ to 10^0^) and cost (range: 10^0^ to 10^3^) parameters.
AdaBoost: Parameters were tuned via an error minimization loop, setting mfinal(number of boosting iterations) between 1 and (number of features - 1), with a maxdepth of 5.
RF: Fixed parameter methodology based on preliminary experiments was used, setting mtry to 1 and ntree to 1000.
LR: The model was built using default settings without regularization, and the classification threshold was adjusted to 0.34.
DT: Pre-pruning was applied by testing the complexity parameter cp, which was finally set to 0.015.”

Univariate analysis and LASSO regression were also used to select clinically significant pathological and conventional ultrasound features associated with CSI in EC, forming a clinical model. Univariate analysis was initially applied to reduce the dimensionality of the clinical features, filtering out variables with non-significant P-values (*p* ≥ 0.05). Subsequently, LASSO regression was employed on the remaining features to identify the most significant non-zero coefficients and eliminate redundant features to prevent overfitting. As shown in **Supplementary Figure S1**, this process shrunk the coefficients of irrelevant features to zero, resulting in the final six key features used to build the logistic regression model (Details of the univariate analysis are provided in **Supplementary Table S1**). Radiomic scores (Radscore) were calculated based on important radiomic features identified through LASSO regression, while clinical scores (C_score) were derived from significant clinical features. A nomogram model was then constructed by integrating both Radscore and C_score (The calculation formulas were provided in the **Supplementary Materials**) using multivariate logistic regression.

### 2.7 Statistical methods

Statistical analyses were performed using Python (version 3.11.4) and R (version 4.4.2). Continuous variables were expressed as mean ± standard deviation (x̅ ± s). Data distribution was assessed using the Shapiro-Wilk normality test. For normally distributed data with homogeneity of variance, independent samples t-tests were used; otherwise, Mann-Whitney U tests were applied. Categorical variables were presented as counts (percentages) and analyzed using chi-square tests or Fisher’s exact test as appropriate. The diagnostic performance of the models was evaluated using receiver operating characteristic (ROC) curves, with area under the curve (AUC), accuracy, sensitivity, specificity, positive predictive value (PPV), negative predictive value (NPV), and F1 score calculated. Differences in AUC between models were compared using DeLong’s test. Calibration curves were plotted to assess the calibration performance of the combined model. Decision curve analysis (DCA) was employed to evaluate the clinical utility of the models. P-value < 0.05 was considered statistically significant.

## 3 Results

### 3.1 Clinical characteristics

The 294 patients were randomly divided into a training cohort (n = 205) and a test cohort (n = 89) at a 7:3 ratio. The comparison of clinical, pathological, and ultrasound characteristics between the training and test cohorts is presented in **Supplementary Table S1**. To prevent data leakage, the screening of all clinical features was strictly performed within the training set. Univariate analysis of the 42 features in the training set indicated that 33 indicators—including vaginal bleeding and discharge, menstrual disorder, abdominal pain, bleeding after intercourse, others, pelvic inflammatory disease, atherosclerosis, hypertension, diabetes, hyperlipidemia, fatty liver, heart disease, history of previous cesarean section, history of ectopic pregnancy, history of previous uterine surgery, age at menarche, age at menopause, age at marriage, gravida, abortions or induced labor instances, weight, height, body mass index(BMI), carcinoembryonic antigen (CEA), squamous cell carcinoma antigen (SCC-Ag), cytokeratin 19 fragment (CYFRA 21-1), human papillomavirus (HPV), uterine volume, uterine shape, intrauterine fluid accumulation, tumor echogenicity, tumor echogenicity intensity, and endometrial thickness—were not statistically significant (*p* ≥ 0.05). Conversely, 9 indicators—age, duration of menopause, menopausal status, parity, CA-125, maximum tumor diameter, tumor shape, endometrial-myometrial junction integrity, and tumor vascularity grade—showed statistical significance (*p* < 0.05) and were thus selected for the subsequent LASSO regression analysis. Subsequently, LASSO regression analysis identified six features with non-zero coefficients: duration of menopause, parity, CA-125, maximum tumor diameter, endometrial-myometrial junction integrity, and tumor vascularity grade (**Supplementary Figure S1**).

### 3.2 Radiomics model development

**Figure 2:**
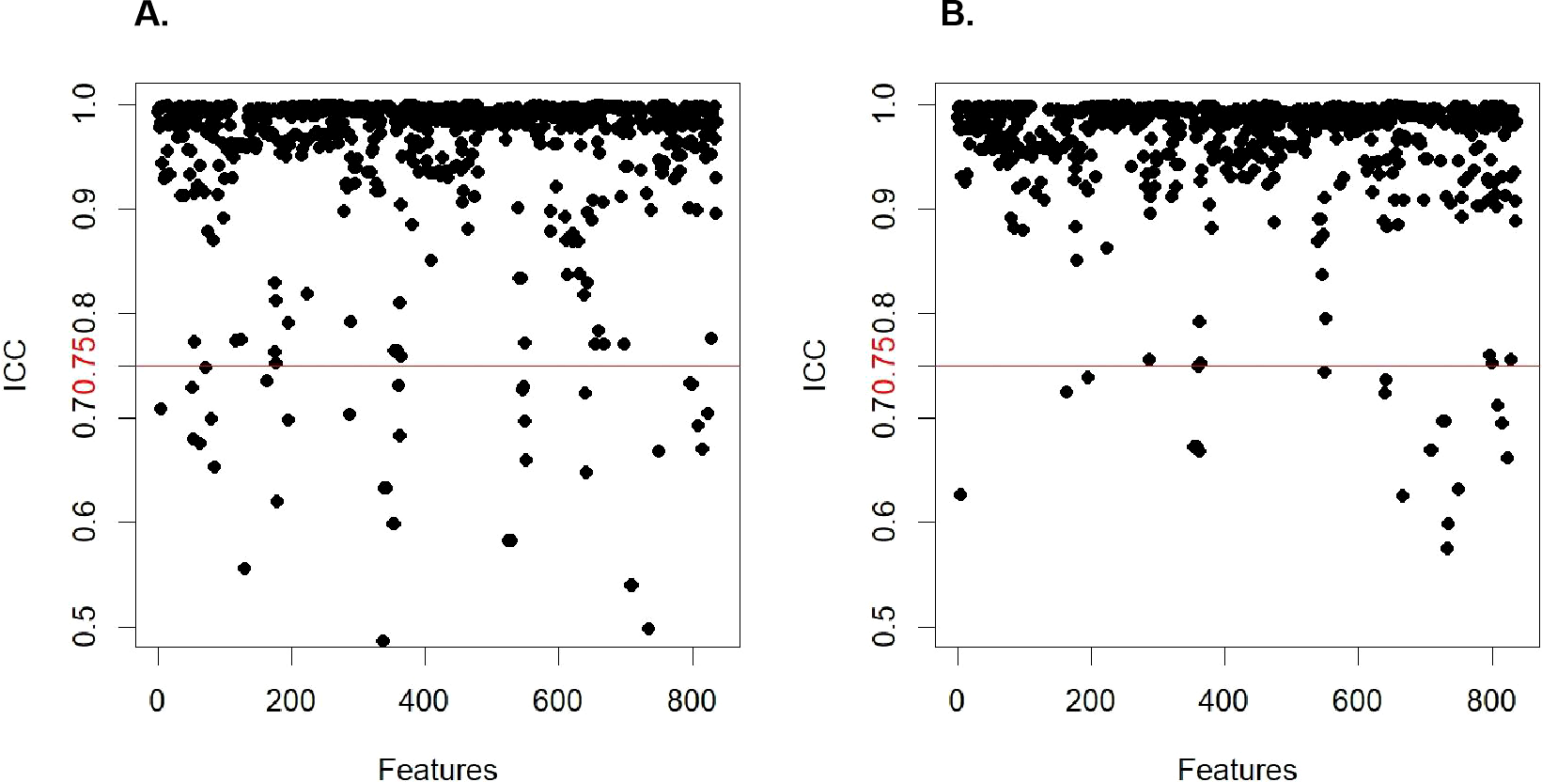
Intraclass and interclass correlation coefficient distribution plots. **(A)** Intraclass correlation coefficient; **(B)** Interclass correlation coefficient.

A total of 837 radiomic features were extracted for each patient, comprising 162 first-order statistics and 675 texture features. The texture features included 216 from the gray-level co-occurrence matrix (GLCM), 126 from the gray-level dependence matrix (GLDM), 144 from the gray-level run length matrix (GLRLM), 144 from the gray-level size zone matrix (GLSZM), and 45 from the neighboring gray tone difference matrix (NGTDM). Of these, 783 features demonstrated high reproducibility with ICCs ≥ 0.75 (**Figure 2**). A total of 260 features were retained after univariate analysis. Following LASSO feature selection, 25 features were retained—20 texture features and 5 first-order features (**Supplementary Figure S1**). Among all radiomics models constructed using different ML algorithms, the LR model achieved the best performance, with AUC values of 0.975 (95% CI: 0.958–0.991) in the training cohort and 0.906 (95% CI: 0.848–0.965) in the test cohort. The model also showed favorable performance in terms of sensitivity, specificity, accuracy, PPV, NPV, and F1 score: in the training cohort, these metrics were 0.940, 0.885, 0.848, 0.956, 0.907, and 0.891, respectively; in the test cohort, they were 0.917, 0.755, 0.717, 0.930, 0.820, and 0.805, respectively (**Table 1**). Therefore, the LR model was selected for subsequent model development.

**Table 1:** Comparison of the Performance of Five Radiomics Models.

group	model	AUC(95%CI)	sensitivity	specificity	PPV	NPV	accuracy	F1 score
training	LR	0.975(0.958-0.991)	0.940	0.885	0.848	0.956	0.907	0.891
	DT	0.972(0.953-0.990)	0.904	0.926	0.893	0.934	0.917	0.898
	RF	0.991(0.984-0.998)	0.976	0.934	0.910	0.983	0.951	0.942
	SVM	1.000	1.000	1.000	1.000	1.000	1.000	1.000
	AdaBoost	0.957(0.933-0.980)	0.880	0.877	0.830	0.915	0.878	0.854
test	LR	0.906(0.848-0.965)	0.917	0.755	0.717	0.930	0.820	0.805
	DT	0.708(0.592-0.824)	0.722	0.698	0.619	0.787	0.708	0.667
	RF	0.726(0.648-0.865)	0.611	0.736	0.611	0.736	0.685	0.611
	SVM	0.833(0.750-0.917)	0.722	0.755	0.667	0.800	0.742	0.693
	AdaBoost	0.750(0.650-0.851)	0.694	0.642	0.568	0.756	0.663	0.625

LR, logistic regression; SVM, support vector machines; RF, random forests; AdaBoost, gradient boosting; DT, decision tree.s

### 3.3 Clinical model development

**Figure 3:**
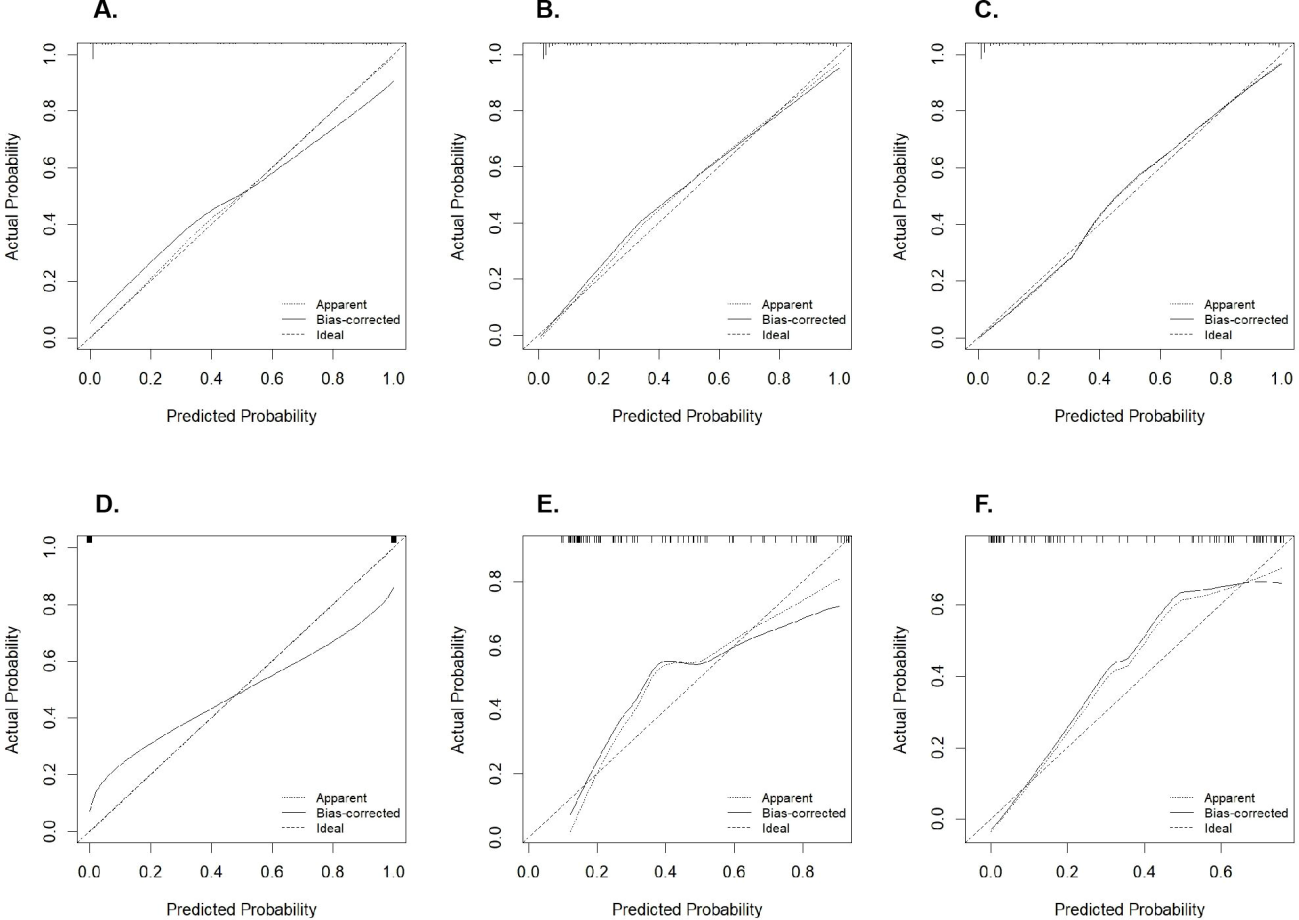
ROC curves of the various models. **(A)** ROC curve of the radiomics model in the training cohort; **(B)** ROC curve of the clinical model in the training cohort; **(C)** ROC curve of the nomogram model in the training cohort; **(D)** ROC curve of the radiomics model in the test cohort; **(E)** ROC curve of the clinical model in the test cohort; **(F)** ROC curve of the nomogram model in the test cohort; AUC, the area under the receiver operating characteristic curve; LR, logistic regression; ROC, the receiver operating characteristic curve.

In the training cohort, age, duration of menopause, menopausal status, parity, CA-125, maximum tumor diameter, tumor shape, endometrial-myometrial junction integrity, and tumor vascularity grade showed significant differences between the CSI and non-invasion groups (**Supplementary Table S1**, *p* < 0.05). Subsequently, LASSO regression analysis identified six features with non-zero coefficients: duration of menopause, parity, CA-125, maximum tumor diameter, endometrial-myometrial junction integrity, and tumor vascularity grade (**Supplementary Figure S1**). Based on these six features, a clinical model was constructed using LR. The model achieved AUC values of 0.947 (95% CI: 0.920–0.974) in the training cohort and 0.832 (95% CI: 0.749–0.915) in the test cohort. Performance metrics in the training cohort were: sensitivity 0.867, specificity 0.869, PPV 0.818, NPV 0.906, accuracy 0.868, and F1 score 0.842; in the test cohort: sensitivity 0.722, specificity 0.736, PPV 0.650, NPV 0.800, accuracy 0.730, and F1 score 0.684 (**Figure 3**). DeLong tests revealed that the difference in AUC between the clinical model and the radiomics model was statistically significant in both the training cohort (Z = 2.326, *p* = 0.020) and the test cohort (Z = 2.371, *p* = 0.018) (**Supplementary Figure S2**).

### 3.4 Nomogram model construction

**Figure 4:**
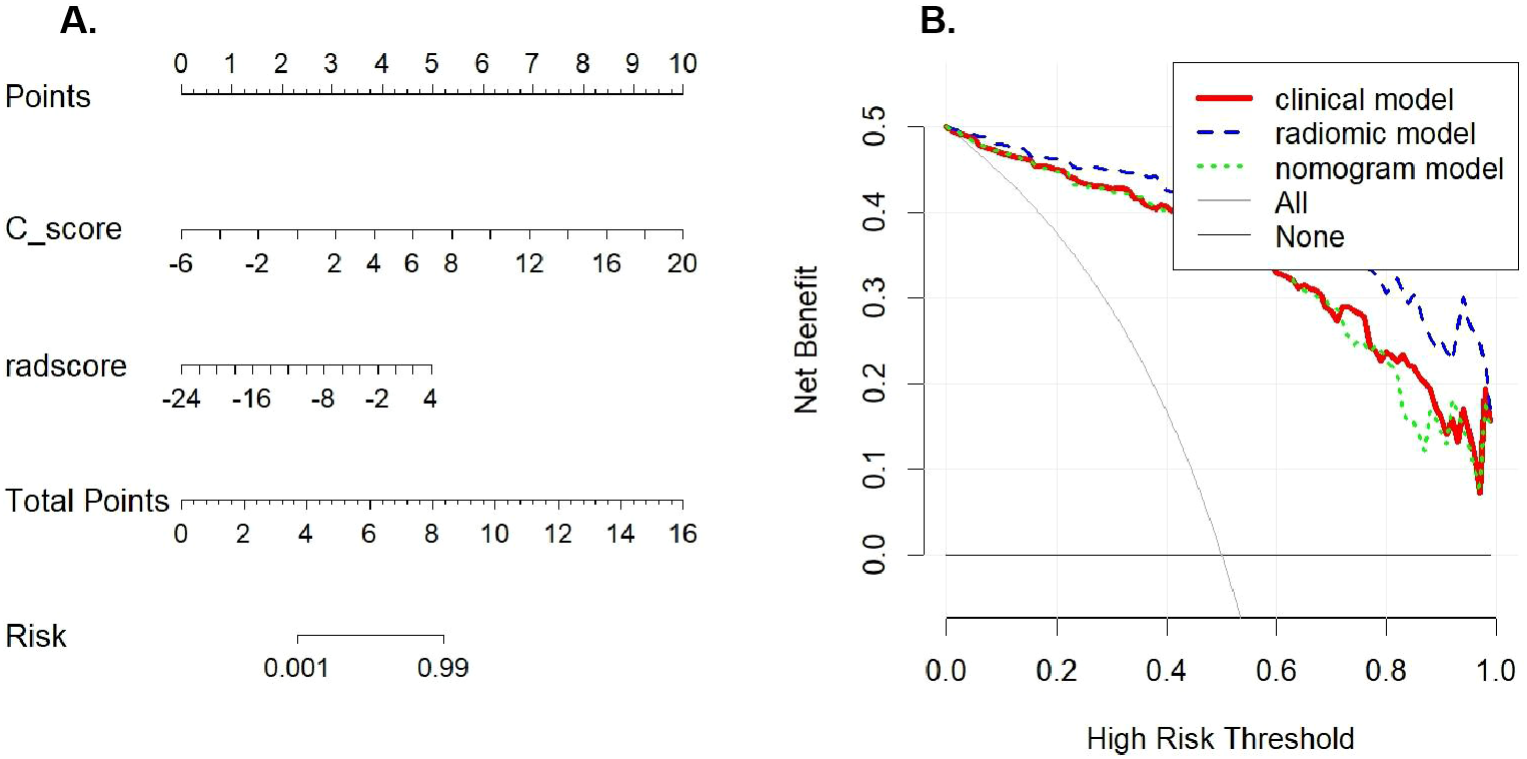
The nomogram and decision curve analysis. **(A)** The nomogram of the model combining radscore and C_score; **(B)** decision curve analysis of the ultrasound radiomics model, clinical model, and nomogram model.

Using both the Radscore and C_score (The calculation formulas were provided in the **Supplementary Materials**), a nomogram model was constructed via multivariate LR (**Figure 4**). The performance metrics of this nomogram model in the training cohort were: AUC of 0.945 (95% CI: 0.917–0.974), sensitivity 0.928, specificity 0.828, PPV 0.786, NPV 0.944, accuracy 0.868, and F1 score 0.851. In the test cohort, the metrics were: AUC of 0.838 (95% CI: 0.756–0.919), sensitivity 0.722, specificity 0.736, PPV 0.650, NPV 0.796, accuracy 0.730, and F1 score 0.684. DCA demonstrated that all three models (radiomics, clinical, and nomogram) provided higher net clinical benefit compared to treating all or none within a threshold probability range of 0-1, indicating substantial clinical utility across this range (**Figure 4**).

**Figure 5:**
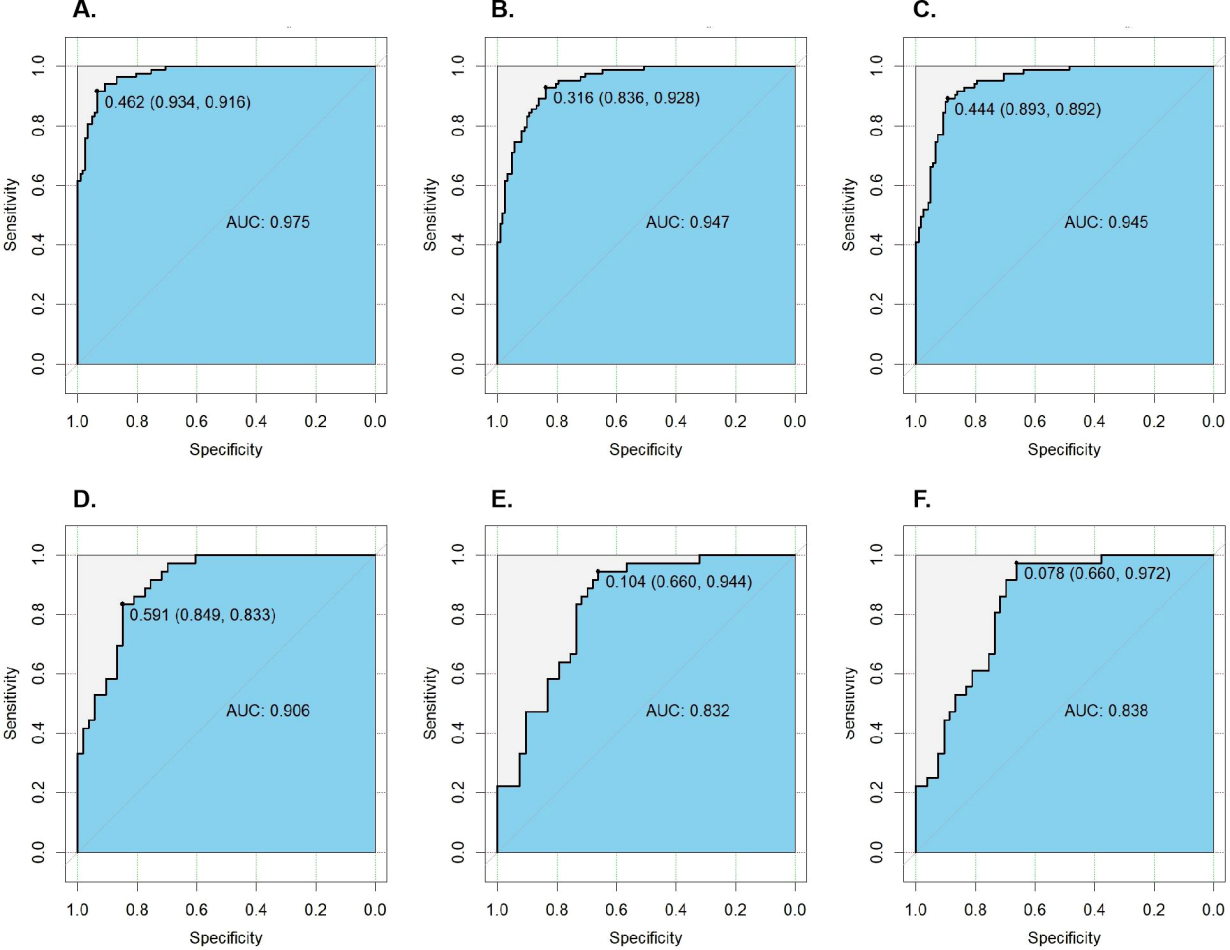
Calibration curves of the models. **(A)** Calibration curve of the radiomics model in the training cohort; Figure **(B)** Calibration curve of the clinical model in the training cohort; **(C)** Calibration curve of the nomogram model in the training cohort; **(D)** Calibration curve of the radiomics model in the test cohort; **(E)** Calibration curve of the clinical model in the test cohort; **(F)** Calibration curve of the nomogram model in the test cohort.

DeLong tests showed no significant difference in AUC between the nomogram model and the clinical model in the training cohort (Z = 0.513, *p* = 0.607) or in the test cohort (Z = 1.103, *p* = 0.268) (**Supplementary Figure S2**). However, there was a significant difference in AUC between the nomogram model and the radiomics model in the training cohort (Z = 2.053, *p* = 0.012; **Supplementary Figure S2**), as well as in the test cohort (Z = 2.261, *p* = 0.024; **Supplementary Figure S2**). Calibration curves indicated excellent agreement between predicted and observed values for all three models in both the training and test cohorts (**Figure 5**).

## 4 Discussion

CSI is a critical factor in the staging and risk stratification of EC, providing important guidance for clinical diagnosis and treatment (6, 10). In recent years, radiomics has been extensively applied in the study of EC (24, 25). Our study demonstrates that radiomic features derived from tumor ultrasound images are closely associated with CSI in EC. The predictive models based on radiomics can non-invasively predict the presence of CSI prior to treatment.

A total of 837 radiomic features were extracted in this study. After rigorous screening, 25 highly reproducible features were retained, and a radiomics model was constructed using LR, which demonstrated optimal performance. The model achieved AUC values of 0.975 in the training cohort and 0.906 in the test cohort, indicating robust diagnostic accuracy. In comparison, Wang X et al. (22) developed a model based on MRI to predict CSI in EC, reporting an AUC of 0.924 in the training set and AUCs of 0.823 and 0.806 in two independent validation sets. Our model outperformed theirs in both cohorts. The performance of our best model is comparable to that of a recent study combining radiomics with three-dimensional deep transfer learning on multiparametric MRI for predicting CSI in EC patients (23). These results suggest that ultrasound-based radiomics models can effectively capture the microscopic heterogeneity of tumors and hold significant value in predicting CSI. Furthermore, ultrasound imaging is more cost-effective, easier to perform, and faster than MRI, offering greater clinical practicality and accessibility.

In the construction of the clinical model, we identified six clinical features significantly associated with CSI through univariate analysis and LASSO regression: duration of menopause, parity, CA-125 level, maximum tumor diameter, status of the endometrial-myometrial junction, and tumor vascularity grade. These indicators have been repeatedly validated in previous studies as being closely linked to the progression and prognosis of EC (26–28), and have also been incorporated into risk prediction models for high-risk EC (29). The clinical model achieved AUC values of 0.947 in the training cohort and 0.832 in the test cohort, indicating a certain degree of predictive capability. However, the difference in AUC compared to the radiomics model was statistically significant (*p* < 0.05), suggesting that radiomic features offer superior performance in identifying CSI. In contrast, Wang X et al. (22) reported a clinical model for predicting CSI in EC with an AUC of 0.898 in the training set and AUCs of 0.570 and 0.540 in two validation sets. Compared to their results, our clinical model demonstrates higher diagnostic accuracy and better generalizability in the test cohort.

To simplify the model and evaluate the contribution of multimodal parameters, we further integrated clinical and radiomic features to construct a nomogram model, which achieved AUCs of 0.945 in the training cohort and 0.838 in the test cohort. In contrast to the study by Wang X et al. (22), the performance of our combined model was slightly lower than that of the radiomics-only model. However, by incorporating both clinical information and imaging characteristics, the nomogram offers a more streamlined clinical application process and improved interpretability. DCA demonstrated that the nomogram provides high net clinical benefit across a wide range of threshold probabilities, indicating strong potential for clinical utility. Furthermore, calibration curves showed excellent agreement between predicted and observed outcomes, further confirming the model’s robustness and reliability. Interestingly, our results indicate that the radiomics-only model outperformed the combined nomogram model. Although the nomogram model (AUC: 0.838) showed a marginal improvement over the clinical model (AUC: 0.832), this difference was not statistically significant (*p* = 0.268, deLong test). Crucially, the radiomics model (AUC: 0.906) demonstrated significantly higher discriminatory power than both the nomogram model (*p* = 0.024, deLong test) and the clinical model (*p* = 0.018, deLong test). These results indicate that the radscore is the dominant predictor in this diagnostic task, and the addition of clinical variables did not enhance the model’s performance in the test cohort. This phenomenon suggests that the high-throughput radiomic features extracted from ultrasound images may capture the pathological heterogeneity of CSI more purely and robustly than the combined clinical-radiological profile. The slight decrease in AUC observed in the nomogram implies that the selected clinical variables, while significant in the training phase, might not be as stable as the radiomic features when generalizing to external populations. Therefore, this study indicates that the radiomics model, rather than the integrated nomogram, represents the optimal tool for the non-invasive prediction of CSI.

Unlike many studies that rely on high-cost or less accessible imaging modalities, our model was developed with a focus on “clinical utility in resource-constrained settings”. By utilizing standard transvaginal ultrasound—a tool available in nearly all gynecological clinics—we offer a solution that is immediately deployable without requiring expensive infrastructure. Furthermore, we emphasize the robustness of our feature selection pipeline. By strictly adhering to reproducibility screening (ICC ≥ 0.75) and following standardized radiomics guidelines, we ensured that the extracted features are not merely statistical artifacts but represent true biological heterogeneity of the tumor. This rigorous methodology enhances the internal validity of our model, providing a solid foundation for future external validation studies. The primary innovation of this work, therefore, lies not just in the AUC value, but in demonstrating that high diagnostic accuracy can be achieved through a non-invasive, low-cost, and widely available technique, filling a critical gap in the preoperative assessment of CSI.

Nevertheless, this study has several limitations. First, as a single-center retrospective study, our research is inherently susceptible to selection bias. Regarding sample size, although our total cohort (n=294) meets the general requirement for radiomics modeling, the number of positive events (patients with cervical stromal invasion, n=119) may still impose limitations on the stability and generalizability of the model. A larger sample size, particularly a higher proportion of positive cases, is typically required to robustly validate complex machine learning algorithms and to ensure the model does not overfit to the specific characteristics of our institution. Consequently, the current model should be considered as preliminary, and its stability needs to be confirmed through external validation. Second, only 2D ultrasound images were used for radiomic analysis. These limitations suggest that future research should expand the sample size, incorporate radiomic analyses from contrast-enhanced ultrasound and three-dimensional ultrasound, and include prospective validation to improve the model’s accuracy and reliability. Such efforts would ensure the effectiveness and broad applicability of the proposed non-invasive method in clinical practice. Additionally, developing artificial intelligence-based automated segmentation techniques in the future will be essential to improve efficiency and reproducibility, thereby facilitating clinical translation and widespread adoption. Furthermore, due to the retrospective nature and single-institution design, our model lacks external validation. It is imperative to emphasize the necessity of external validation through multicenter prospective cohort studies. Such studies are crucial to verify the robustness of the nomogram in diverse clinical settings and to mitigate the impact of institutional-specific biases. Future research should prioritize prospective data collection across multiple centers to establish a universally applicable predictive tool.

It is important to acknowledge the inherent limitations of ultrasound imaging. While ultrasound is the primary modality for initial screening due to its accessibility and real-time capabilities, it is constrained by physical factors such as limited penetration depth and lower soft-tissue resolution compared to magnetic resonance imaging (MRI). Conventional ultrasound relies heavily on morphological features and Doppler flow information, which may sometimes be insufficient for precisely delineating deep stromal invasion. In this context, the future of precision diagnosis lies in multimodal data fusion. While this study focused solely on ultrasound radiomics to validate its incremental value, integrating multiparametric MRI (which offers superior anatomical detail and functional sequences like DWI) or CT data with clinical-radiological features could create a more comprehensive diagnostic ecosystem. Future studies should aim to develop fusion algorithms that combine the real-time microvascular information from ultrasound with the high-resolution structural data from MRI, thereby overcoming the resolution limitations of a single modality. Furthermore, we acknowledge the limitations of traditional hand-crafted radiomics features. While this study successfully utilized conventional radiomics to construct a predictive model, these manually engineered features are often limited to low-dimensional statistical descriptions (e.g., texture and shape) and may fail to capture the complex, high-dimensional patterns hidden within ultrasound images. This constraint can lead to suboptimal performance in heterogeneous clinical environments. To address this, we believe that future iterations of this research should transition towards DL or foundation models. Unlike traditional methods, DL models can automatically extract high-dimensional abstract features directly from raw images, offering superior generalizability and robustness. In resource-limited settings—where standardization of equipment and imaging protocols is challenging—deep learning’s ability to learn invariant features could be pivotal in developing a universally applicable, non-invasive diagnostic tool for CSI.

In conclusion, this study demonstrates that ultrasound-based radiomics models can effectively predict CSI in patients with early-stage EC prior to surgery. Although the nomogram combining C_score and Radscore did not significantly outperform the individual models, both the clinical and nomogram models exhibited strong predictive performance. Notably, the radiomics model itself achieved exceptionally high predictive accuracy. This non-invasive approach holds promise for identifying CSI and may provide novel therapeutic strategies and clinical guidance for patients with early-stage EC.

## Data Availability

The original contributions presented in the study are included in the article/**Supplementary Material**. Further inquiries can be directed to the corresponding author.
