# Supplementary material for: Prediction of cervical stromal invasion using ultrasound radiomics: from conventional ultrasound to intelligent diagnosis
Source: Front Oncol. 2026 May 7;16:1817583. doi: 10.3389/fonc.2026.1817583 (PMC13189963; doi:10.3389/fonc.2026.1817583)
Supplement: Supplementary file 2 [file Table1.docx]

Table S1 Clinical, pathological and ultrasound characteristics for patients with endometrial cancer, included in the training and test set.

| Characteristics | Training set | | | | Test set | | | |
| --- | --- | --- | --- | --- | --- | --- | --- | --- |
|  | CSI  (n=83) | Non-CSI (n=122) | t/Z/χ² | P-value | CSI  (n=36) | Non-CSI (n=53) | t/Z/χ² | P-value |
| Age (years) (mean ± SD) | 52.69±8.04 | 57.02±8.58 | 3501 | <0.001 | 54.26±8.45 | 56.39±9.63 | 843.5 | 0.357 |
| Vaginal bleeding and discharge n(%) |  |  | 0.009 | 0.925 |  |  | 0.733 | 0.392 |
| No | 12 (9.8) | 110 (90.2) |  |  | 9 (17) | 44 (83) |  |  |
| Yes | 7 (8.4) | 76 (91.6) |  |  | 3 (8.3) | 33 (91.7) |  |  |
| Menstrual disorder n(%) |  |  | 2.454 | 0.117 |  |  | 0.388 | 0.533 |
| No | 107 (87.7) | 15 (12.3) |  |  | 45 (84.9) | 8 (15.1) |  |  |
| Yes | 79 (95.2) | 4 (4.8) |  |  | 33 (91.7) | 3 (8.3) |  |  |
| Abdominal pain n(%) |  |  | 0.371 | 0.542 |  |  | 4.007 | 0.045 |
| No | 113 (92.6) | 9 (7.4) |  |  | 50 (94.3) | 3 (5.7) |  |  |
| Yes | 74 (89.2) | 9 (10.8) |  |  | 28 (77.8) | 8(22.2) |  |  |
| Bleeding after intercourse n(%) |  |  | 0.004 | 0.952 |  |  | 0.038 | 0.845 |
| No | 119 (97.5) | 3 (2.5) |  |  | 53 (100) | 0 (0) |  |  |
| Yes | 80 (96.4) | 3 (3.6) |  |  | 35 (97.2) | 1 (2.8) |  |  |
| Others n(%) |  |  | 0.82 | 0.365 |  |  | 0.004 | 0.95 |
| No | 121 (99.2) | 1 (0.8) |  |  | 50 (94.3) | 3 (5.7) |  |  |
| Yes | 80 (96.4) | 3 (3.6) |  |  | 33 (91.7) | 3 (8.3) |  |  |
| Pelvic inflammatory disease n(%) |  |  | 0.269 | 0.604 |  |  | 0.04 | 0.842 |
| No | 111 (91) | 11 (9) |  |  | 49 (92.5) | 4 (7.5) |  |  |
| Yes | 78 (94) | 5 (6) |  |  | 32 (88.9) | 4 (11.1) |  |  |
| Atherosclerosis n(%) |  |  | 1.631 | 0.202 |  |  | 7.864 | 0.005 |
| No | 100 (82) | 22 (18) |  |  | 46 (86.8) | 7 (13.2) |  |  |
| Yes | 61 (73.5) | 22 (26.5) |  |  | 21 (58.3) | 15 (41.7) |  |  |
| Hypertension n(%) |  |  | 0.001 | 0.975 |  |  | 0.009 | 0.923 |
| No | 97 (79.5) | 25 (20.5) |  |  | 44 (83) | 9 (17) |  |  |
| Yes | 65 (78.3) | 18 (21.7) |  |  | 31 (86.1) | 5 (13.9) |  |  |
| Diabetes n(%) |  |  | 0.297 | 0.586 |  |  | 1.879 | 0.17 |
| No | 106 (86.9) | 16 (13.1) |  |  | 48 (90.6) | 5 (9.4) |  |  |
| Yes | 69 (83.1) | 14 (16.9) |  |  | 28 (77.8) | 8 (22.2) |  |  |
| Hyperlipidemia n(%) |  |  | 0.069 | 0.793 |  |  | 0.015 | 0.902 |
| No | 117 (95.9) | 5 (4.1) |  |  | 50 (94.3) | 3 (5.7) |  |  |
| Yes | 81 (97.6) | 2 (2.4) |  |  | 35 (97.2) | 1 (2.8) |  |  |
| Fatty liver n(%) |  |  | 0.061 | 0.805 |  |  | 1.696 | 0.193 |
| No | 88 (72.1) | 34 (27.9) |  |  | 43 (81.1) | 10 (18.9) |  |  |
| Yes | 62 (74.7) | 21 (25.3) |  |  | 24 (66.7) | 12 (33.3) |  |  |
| Heart disease n(%) |  |  | 0 | 1 |  |  | 1.358 | 0.244 |
| No | 120 (98.4) | 2 (1.6) |  |  | 49 (92.5) | 4 (7.5) |  |  |
| Yes | 82 (98.8) | 1 (1.2) |  |  | 36 (100) | 0 (0) |  |  |
| History of previous cesarean section n(%) |  |  | 0.631 | 0.427 |  |  | 0 | 1 |
| No | 115 (94.3) | 7 (5.7) |  |  | 51 (96.2) | 2 (3.8) |  |  |
| Yes | 81 (97.6) | 2 (2.4) |  |  | 35 (97.2) | 1 (2.8) |  |  |
| History of ectopic pregnancy n(%) |  |  | 0.015 | 0.902 |  |  | - | - |
| No | 119 (97.5) | 3 (2.5) |  |  | 53 (100) | 0 (0) |  |  |
| Yes | 82 (98.8) | 1 (1.2) |  |  | 36 (100) | 0 (0) |  |  |
| History of previous uterine surgery n(%) |  |  | 0 | 1 |  |  | 0.729 | 0.393 |
|  | 119 (97.5) | 3 (2.5) |  |  | 50 (94.3) | 36 (5.7) |  |  |
|  | 81 (97.6) | 2 (2.4) |  |  | 36 (100) | 0 (0) |  |  |
| Age at menarche (years) (mean ± SD) | 14.12±2.86 | 14.18±1.47 | 4385.5 | 0.087 | 14.02±1.34 | 14.14±1.42 | 940.5 | 0.908 |
| Age at menopause (years) (mean ± SD) | 49.84±2.59 | 49.69±2.97 | 5034.5 | 0.94 | 49.77±1.99 | 50±2.97 | 908.5 | 0.661 |
| Duration of menopause n(%) | 3.25±5.73 | 6.98±8.35 | 3612 | <0.001 | 3.21±6.7 | 5.78±8.9 | 771 | 0.103 |
| Menopausal status n(%) |  |  | 7.136 | 0.008 |  |  | 0.985 | 0.321 |
| No | 67 (54.9) | 55 (45.1) |  |  | 29 (54.7) | 24 (45.3) |  |  |
| Yes | 29 (34.9) | 54 (65.1) |  |  | 15 (41.7) | 21 (58.3) |  |  |
| Age at marriage (years) (mean ± SD) | 22.16±4.11 | 21.55±2.18 | 5452 | 0.335 | 21.92±2.21 | 21.69±2.21 | 1062.5 | 0.353 |
| Gravida n(%) |  |  | 0.006 | 0.936 |  |  | 0.446 | 0.504 |
| <3 | 46 (37.7) | 76 (62.3) |  |  | 18 (34) | 35 (66) |  |  |
| ≥3 | 30 (36.1) | 53 (63.9) |  |  | 9 (25) | 27 (75) |  |  |
| Parity n(%) |  |  | 5.489 | 0.019 |  |  | 0 | 1 |
| <3 | 112 (91.8) | 10 (8.2) |  |  | 45 (84.9) | 8 (15.1) |  |  |
| ≥3 | 66 (79.5) | 17 (20.5) |  |  | 31 (86.1) | 5 (13.9) |  |  |
| Abortions or induced labor instances n(%) |  |  | 0.689 | 0.406 |  |  | 2.996 | 0.083 |
| <3 | 96 (78.7) | 26 (21.3) |  |  | 46 (86.8) | 7 (13.2) |  |  |
| ≥3 | 70 (84.3) | 13 (15.7) |  |  | 25 (69.4) | 11 (30.6) |  |  |
| Weight (kg) (mean ± SD) | 61.17±9.92 | 59.21±9.57 | 5453 | 0.348 | 59.27±7.11 | 60.97±10.42 | 922 | 0.791 |
| Height (cm) (mean ± SD) | 156.05±4.24 | 155.82±5.37 | 5263.5 | 0.629 | 155.62±4.13 | 155.5±4.81 | 999.5 | 0.704 |
| Body Mass Index (BMI) (Kg/cm^2^) (mean ± SD) | 25.11±3.81 | 24.38±3.78 | 5521 | 0.272 | 24.47±2.76 | 25.18±3.92 | 888 | 0.584 |
| Carcinoembryonic Antigen (CEA) (μg/L) (mean ± SD) | 1.57±2.92 | 1.28±1.83 | 4731.5 | 0.426 | 1.02±0.65 | 2.22±5.93 | 883.5 | 0.558 |
| Cancer Antigen 125 (CA-125) (U/ml) (mean ± SD) | 50.55±126.57 | 141.04±475.54 | 4164 | 0.031 | 69.98±196.36 | 83.78±174.62 | 853 | 0.401 |
| Squamous Cell Carcinoma Antigen (SCC-Ag) (ng/ml) (mean ± SD) | 1.49±3.24 | 1.12±0.78 | 5490.5 | 0.295 | 1.03±0.68 | 5.05±18.4 | 738 | 0.065 |
| Cytokeratin 19 Fragment (CYFRA 21-1) (ng/ml) (mean ± SD) | 3.58±9.71 | 2.69±1.21 | 5178.5 | 0.777 | 5.2±19.6 | 3.3±1.67 | 761.5 | 0.098 |
| Human Papillomavirus (HPV) n(%) |  |  | 0 | 1 |  |  | 0.071 | 0.79 |
| No | 114 (93.4) | 8 (6.6) |  |  | 48 (90.6) | 5 (9.4) |  |  |
| Yes | 78 (94) | 5 (6) |  |  | 34 (94.4) | 2 (5.6) |  |  |
| Uterine volume (mm³) (mean ± SD) | 113.56±80.17 | 117.5±113.03 | 5184 | 0.773 | 109.71±69.17 | 138.22±116.24 | 830.5 | 0.304 |
| Uterine shape n(%) |  |  | 0.001 | 0.98 |  |  | 0.314 | 0.575 |
| Regular | 72 (59) | 50 (41) |  |  | 28 (52.8) | 25 (47.2) |  |  |
| Irregular | 48 (57.8) | 35 (42.2) |  |  | 16 (44.4) | 20 (55.6) |  |  |
| Intrauterine fluid accumulation n(%) |  |  | 0.192 | 0.661 |  |  | 0.203 | 0.653 |
| No | 120 (98.4) | 2 (1.6) |  |  | 51 (96.2) | 2 (3.8) |  |  |
| Yes | 80 (96.4) | 3 (3.6) |  |  | 36 (100) | 0 (0) |  |  |
| Maximum tumor diameter (mm) (mean ± SD) | 14.68±10.08 | 43.83±20.22 | 648.5 | <0.001 | 18.34±17.24 | 41.19±21.63 | 313.5 | <0.001 |
| Tumor echogenicity n(%) |  |  | 0.794 | 0.373 |  |  | 0.329 | 0.567 |
| Homogeneous | 47 (38.5) | 75 (61.5) |  |  | 22 (41.5) | 31 (58.5) |  |  |
| Inhomogeneous | 38 (45.8) | 45 (54.2) |  |  | 18 (50) | 18 (50) |  |  |
| Tumor echogenicity intensity n(%) |  |  | 5239.5 | 0.648 |  |  | 817.5 | 0.222 |
| **Hypoechoic** | 19 (15.6) | 17 (20.5) |  |  | 14 (26.4) | 7 (19.4) |  |  |
| **Isoechoic** | 49 (40.2) | 30 (36.1) |  |  | 25 (47.2) | 15 (41.7) |  |  |
| **Hyperechoic** | 54 (44.3) | 36 (43.4) |  |  | 14 (26.4) | 14 (38.9) |  |  |
| Tumor shape n(%) |  |  | 4.345 | 0.037 |  |  | 2.758 | 0.097 |
| Regular | 46 (37.7) | 76 (62.3) |  |  | 22 (41.5) | 31 (58.5) |  |  |
| Irregular | 19 (22.9) | 64 (77.1) |  |  | 8 (22.2) | 28 (77.8) |  |  |
| Endometrium-myometrium junction n(%) |  |  | 4.096 | 0.043 |  |  | 1.576 | 0.209 |
| Regular | 37 (30.3) | 85 (69.7) |  |  | 18(34) | 35 (66) |  |  |
| Irregular | 14 (16.9) | 69 (83.1) |  |  | 7 (19.4) | 29 (80.6) |  |  |
| Tumor blood flow signal intensity n(%) |  |  | 8.475 | 0.004 |  |  | 3.406 | 0.065 |
| Poor | 95 (77.9) | 27 (22.1) |  |  | 43(81.1) | 10(18.9) |  |  |
| Rich | 48 (57.8) | 35 (42.2) |  |  | 22 (61.1) | 14 (38.9) |  |  |
| Endometrial thickness (mm) (mean ± SD) | 3.71±1.52 | 3.73±1.7 | 5039 | 0.942 | 3.72±1.4 | 4.1±2.07 | 887 | 0.504 |

Abbreviations: Results are presented as n (%) or mean (SD). P-values denote the statistical significance of differences between endometrial cancers according to the group.

Figure S1. Radiomics and clinical feature selection using LASSO.


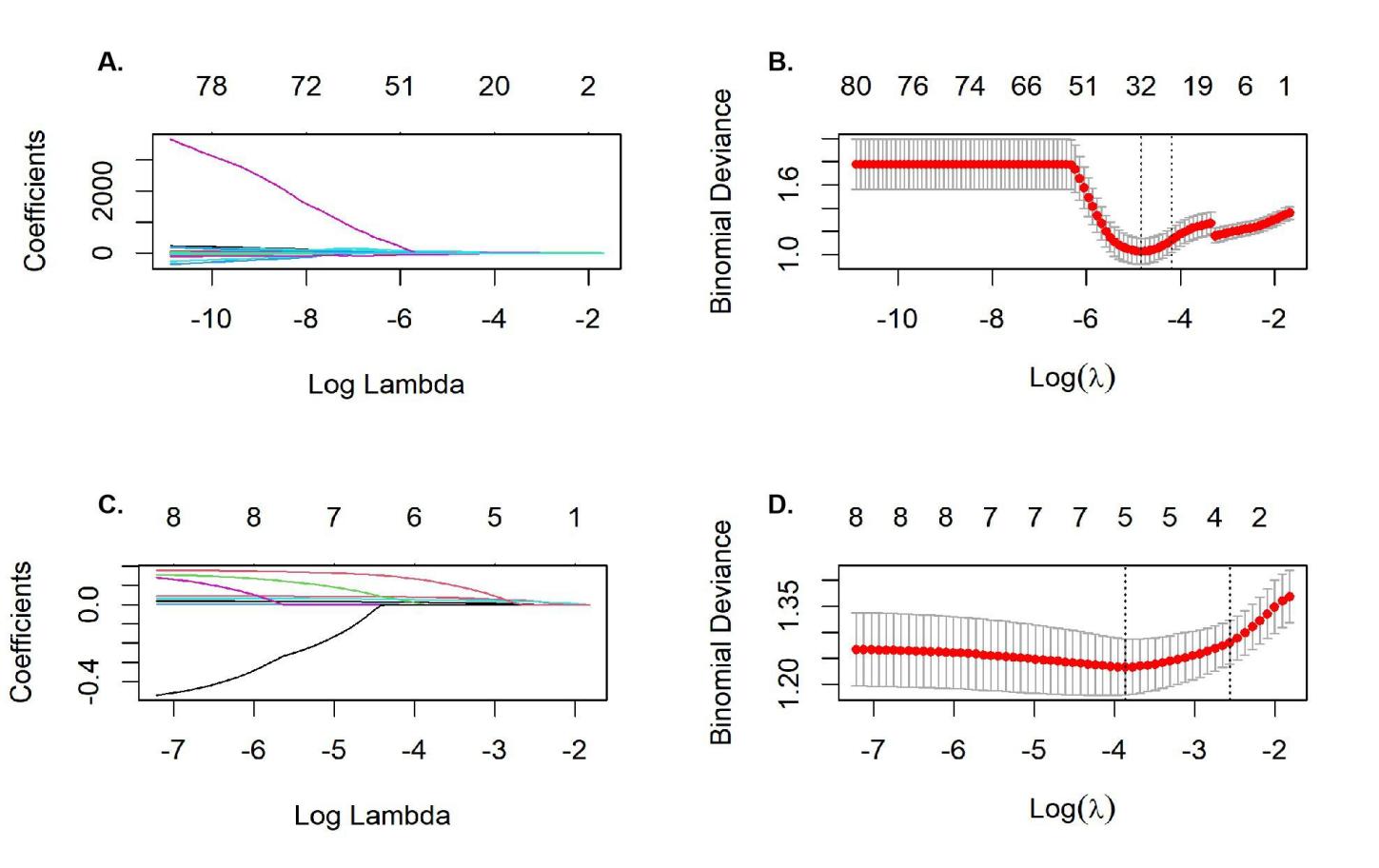


Abbreviations: A. Trajectory of LASSO feature coefficients for radiomics features; B. Radiomics feature screening process diagram; C. Trajectory of LASSO feature coefficients for clinical features; D. Clinical feature screening process diagram.

The calculation formulas for the Radscore and C_score are as follows:

**Radscore** = -16.30868383.

+original_firstorder_MeanAbsoluteDeviation*(-0.047603932).

+original_firstorder_Uniformity*(-0.044482887).

+original_glcm_Contrast*0.939546654070602.

+original_gldm_GrayLevelNonUniformity*2.23867568383426.

+original_gldm_SmallDependenceEmphasis*(-0.231037709).

+original_glrlm_LongRunEmphasis*(-0.510264883).

+original_glszm_GrayLevelVariance*0.0356520754336444.

+wavelet-LLL_glcm_MCC*4.14667924623376.

+original_glszm_ZoneEntropy*(-0.014371763).

+wavelet-LLH_glrlm_GrayLevelNonUniformity*20.7340215644918.

+wavelet-LLH_glrlm_RunVariance*(-1.062494018).

+wavelet-LLH_glszm_LargeAreaHighGrayLevelEmphasis*(-13.30180767).

+wavelet-LLH_glszm_LowGrayLevelZoneEmphasis*0.0765863190944287.

+wavelet-LLH_glszm_ZoneVariance*5.82104889127556.

+wavelet-LHL_ngtdm_Complexity*(-0.161329565).

+wavelet-HLL_glcm_ClusterProminence*(-0.364043275).

+wavelet-HLL_glcm_JointAverage*0.331933487828002.

+wavelet-HLL_glcm_SumEntropy*0.0219851180496553.

+wavelet-HLH_firstorder_Uniformity*0.0602045356663089.

+wavelet-HLH_glcm_DifferenceEntropy*(-0.363382432).

+wavelet-HHL_firstorder_90Percentile*(-2.354523639).

+wavelet-HHL_glcm_Imc2*0.978234490625242.

+wavelet-HHL_glcm_JointEnergy*(-1.574740002).

+wavelet-HHH_firstorder_MeanAbsoluteDeviation*0.00402106536515243.

+wavelet-HHH_glcm_Imc2*(-0.025168983).

**C_score** = -4.732521307.

+Duration of menopause*0.0304052619588447.

+Parity*0.78133750538852.

+CA-125*0.000947197147944821.

+Maximum tumor diameter*0.159405407594143.

+Endometrial-myometrial junction*(-0.530881045).

+Grading of tumor vascularity*0.675491299816903.

Figure S2 DeLong tests of the various models


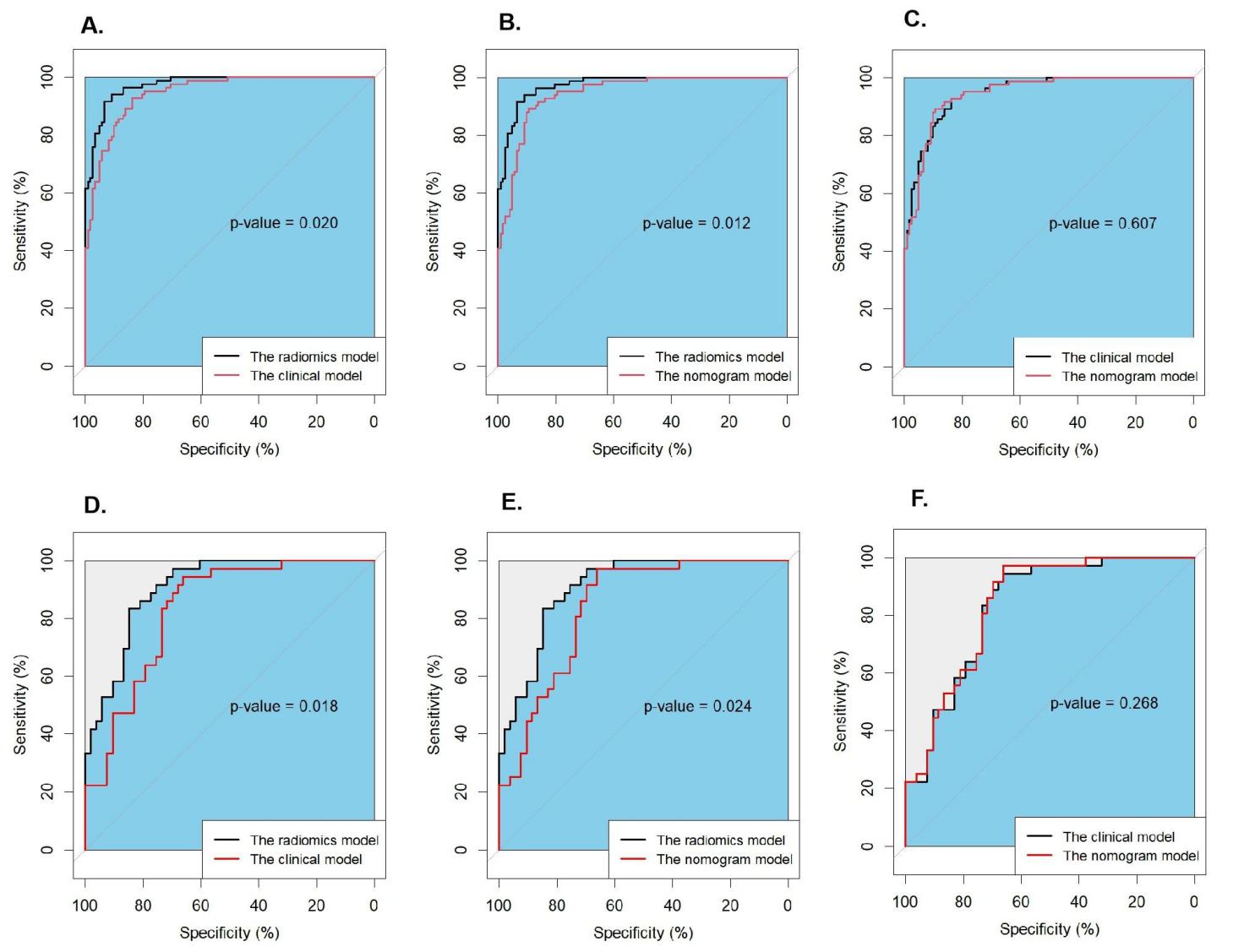


Abbreviations: A. DeLong test of the clinical model and radiomics model in the training cohort; B. DeLong test of the radiomics model and nomogram model in the training cohort; C. DeLong test of the clinical model and nomogram model in the training cohort; D. DeLong test of the clinical model and radiomics model in the test cohort; E. DeLong test of the radiomics model and nomogram model in the test cohort; F. DeLong test of the clinical model and nomogram model in the test cohort.
